# Distribution and functional perspective analysis of epiphytic and endophytic bacterial communities associated with marine seaweeds, Alexandria shores, Egypt

**DOI:** 10.1186/s12866-024-03426-x

**Published:** 2024-08-06

**Authors:** Hanan M. Abdelrazek, Nihal G. Shams El-Din, Hanan A. Ghozlan, Soraya A. Sabry, Samia S. Abouelkheir

**Affiliations:** 1 Faculty of Science, Alexandria University, Moharrem Bey, Alexandria, 21511 Egypt; 2 National Institute of Oceanography and Fisheries (NIOF), Alexandria, Egypt

**Keywords:** Epiphytic bacteria, Endophytic bacteria, Marine seaweeds, Principal components analysis

## Abstract

There is an enormous diversity of life forms present in the extremely intricate marine environment. The growth and development of seaweeds in this particular environment are controlled by the bacteria that settle on their surfaces and generate a diverse range of inorganic and organic chemicals. The purpose of this work was to identify epiphytic and endophytic bacterial populations associated with ten common marine macroalgae from various areas along the Mediterranean Sea coast in Alexandria. This was done to target their distribution and possible functional aspects. Examine the effects of the algal habitat on the counting and phenotypic characterization of bacteria, which involves grouping bacteria based on characteristics such as shape, colour, mucoid nature, type of Gram stain, and their ability to generate spores. Furthermore, studying the physiological traits of the isolates under exploration provides insight into the optimum environmental circumstances for bacteria associated with the formation of algae. The majority of the bacterial isolates exhibited a wide range of enzyme activities, with cellulase, alginase, and caseinase being the most prevalent, according to the data. Nevertheless, 26% of the isolates displayed amylolytic activity, while certain isolates from Miami, Eastern Harbor, and Montaza lacked catalase activity. Geographical variations with the addition of algal extract may impact on the enumeration of the bacterial population, and this might have a relationship with host phylogeny. The most significant observation was that endophytic bacteria associated with green algae increased in all sites, while those associated with red algae increased in Abu Qir and Miami sites and decreased in Eastern Harbor. At the species level, the addition of algal extract led to a ninefold increase in the estimated number of epiphytic bacteria for *Cladophora pellucida* in Montaza. Notably, after adding algal extract, the number of presented endophytic bacteria associated with *Codium* sp. increased in Abu Qir while decreasing with the same species in Montaza. In addition to having the most different varieties of algae, Abu Qir has the most different bacterial isolates.

## Introduction

Seaweeds, also known as marine macroalgae, are photosynthetic nonflowering plant-like organisms classified into three main groups depending on their dominant pigmentation: brown (Phaeophyceae, about 1755 species), red (Rhodophyta, about 6000 species), and green algae (Chlorophyta, about 1500 species) [1, 2]. The industry uses from 7.5 to 8 million metric tons of wet seaweed annually for multi-purpose uses in pharmaceutical industries, agriculture fertilizer, feed for aquaculture or human food additives, which have successfully conquered the market on account of the consumer preference for healthy food.

Marine macrophytes (macroalgae and seagrass) and their epiphytic microbes play an important role in coastal benthic communities. These microbes provide the potential for mutualistic interspecific associations, and they are considered primary producers in the food chain [3]. They enter the second level of the food chain when they are grazed by invertebrates. Ibrahim et al. [4] reported that the production of epiphytic algae often exceeded that of macroalgae and seagrass itself. One of the disadvantages of growing these epiphytes on seaweed is that they cover the photosynthetic area of the seaweed blade, reducing the host’s photosynthetic capabilities. Another disadvantage of epiphytes is that they usually have negative effects on the basiphyte by reducing light availability, impeding gas and nutrient exchange with the water column. According to Brodersen et al. [5], excessive nutrient levels typically induce the rapid proliferation of epiphytes, such as diatoms, which are considered the most essential structural parts of the epiphyton on seaweeds or seagrass.

Algae-bacteria interactions cover the whole range of symbiotic relationships, which are mainly identified as mutualism, commensalism, and parasitism [6]. In addition to spatial variability, epiphytic bacterial community compositions are also driven by differences in seasonal, environmental, and physiological characteristics including elemental composition and nutrient availability [7]. In addition, microbes produce a biofilm matrix consisting of proteins, extracellular DNA, and polysaccharides that is integral to the formation of bacterial communities [8]. It is known that bacteria associated with marine macroalgae offer several important advantages. One of these advantages is the protection against pathogens. Bacteria can produce antimicrobial substances that help protect macroalgae from pathogen infections [9]. For example, Hmani et al. [10] evaluated nine *Ulva ohnoi*-associated bacteria for their positive antimicrobial activity against seven pathogenic bacteria, including *Escherichia coli*, *Vibrio anguillarum*, *Vibrio alginoliticus*, *Pseudomonas aeruginosa*, *Aeromonas hydrophila*, *Salmonella typhymurium*, and *Staphylococcus aureus*, as well as one yeast, *Candida albicans*. Some bacteria can help break down complex organic compounds present in the algae’s environment into simpler, directly usable substances, thus promoting their growth and development. Selvarajan et al.‘s published study [7] reveals that algal organic exudates and elemental deposits significantly influence the diversity of epiphytic bacteria on seaweed surfaces, triggering chemotaxis responses to metabolize substrates. Certain types of bacteria possess the ability to fix atmospheric nitrogen, serving as a nitrogen source for algae in environments where this element is scarce [4]. Meanwhile, other bacteria generate a variety of bioactive compounds that could potentially benefit macroalgae by acting as growth factors or defense agents against herbivores [8]. Bacteria can help stabilize the local environment by forming biofilms on algae surfaces, preventing erosion and promoting sediment fixation [9]. The most important advantage for bacteria in marine biofilms is, probably, access to these resources as carbon and energy sources, micronutrients, and electron donors/acceptors [9]. However, biofilms also protect individual cells against environmental stress, including desiccation, temperature and pH changes, competition and predation, UV exposure, and depleted nutrient conditions [11].

As a consequence, the goal of this study was to isolate and investigate bacterial communities attached to marine algal surfaces in seawater, precisely in Alexandria, Mediterranean Sea, at four distinct sites: Abu Qir, Montaza, Miami, and Eastern Harbor. It was thus aimed at studying the distribution and functional perspective of epiphytic and endophytic bacterial communities associated with marine algae.

## Methods

### Study area

**Fig. 1:**
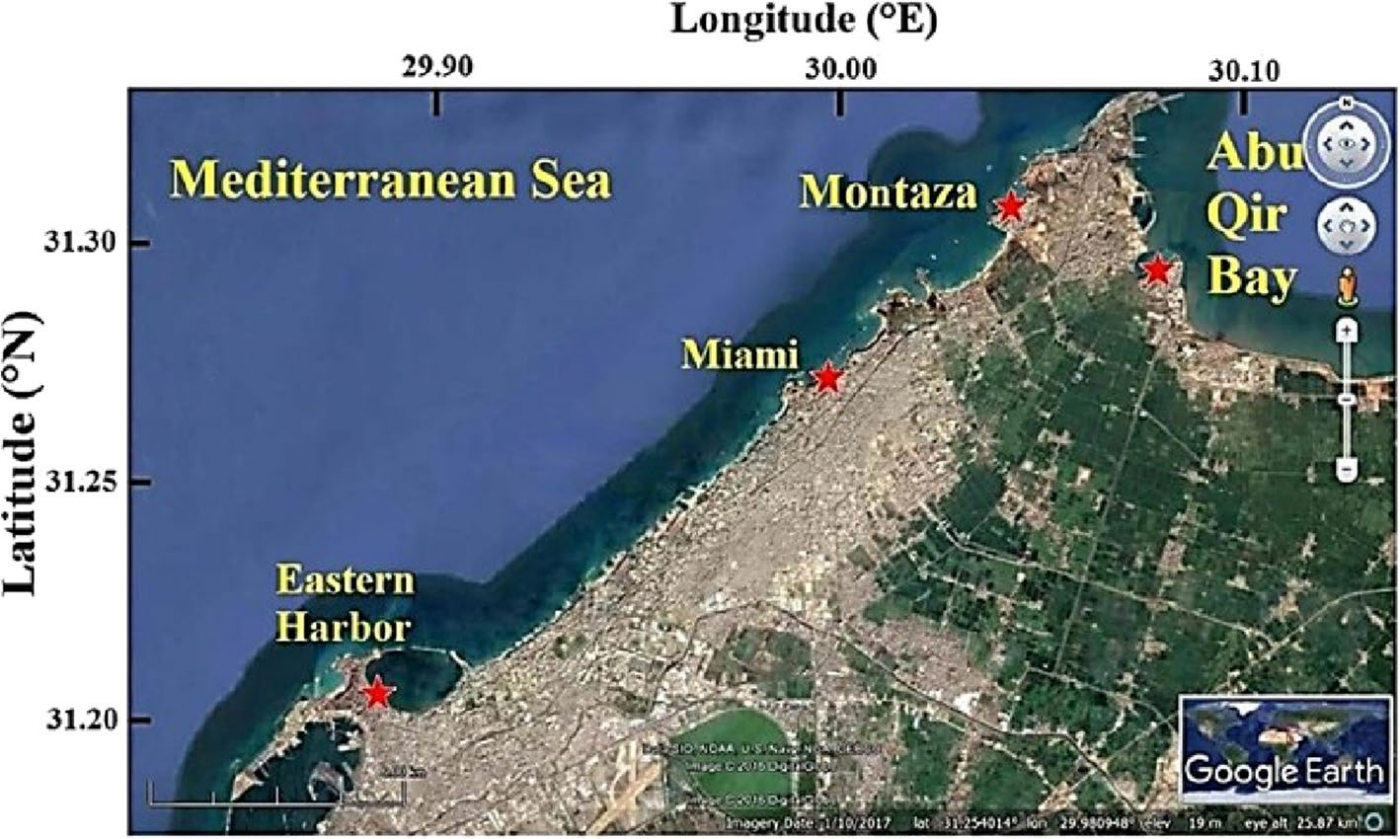
Geographical map of Alexandria shores showing sites of collected algal samples [12]

Algal samples were collected from four stations located in Alexandria, Egypt, along the Mediterranean Sea shores. These stations are Abu Qir (I) (31°19ˋ26ˋˋN, 30°3ˋ41ˋˋE), Montaza (II) (31°10’43.932’’N, 29°49’25.176’’E), Miami (III) (31°16ˋ14ˋˋN, 29°59ˋ36ˋˋE), and Eastern Harbour (IV) (31°12ˋ20ˋˋN, 29°53ˋ1ˋˋE) (Fig. 1) [12].

### Collection of algal samples

Marine algae were collected from the four sites, and the samples were immediately brought to the laboratory in ice boxes for bacterial isolation and enumeration. We then preserved the algae samples in formalin solution (4%) according to Maggs & Abbott [13] for identification. According to Aleem [14] and Cabioch et al. [15], the algae were re-identified by morphological features, utilizing guides and keys for identification. Furthermore, the macroalgal species that were recorded were updated in accordance with the algaebase.com taxonomy database [16].

### Media used for bacterial isolation

#### Nutrient broth (NB) medium (Neogen Culture Media, LAB M, UK)

This medium is composed of (g/l): Peptone, 10, Yeast extract, 2, Beef extract, 3, Sodium chloride, 5. To prepare the solid medium, agar (B&V Laboratory Chemicals, Italy) was added at a concentration of 20 g/l. The pH was adjusted to 7 ± 0.2. The medium was prepared with seawater when required and sterilized by autoclaving at 121 ^°^C for 20 min.

### Isolation of marine algal epiphytes and endophytes

#### Epiphytic bacteria

To isolate epiphytic bacteria, one square centimeter of tissue from each sample was swabbed or rubbed using a wet sterile cotton swab, then inoculated into nutrient broth dissolved in seawater with a serial dilution series. After serial dilution, the aliquots of the diluted sample are plated on nutrient agar that was prepared using seawater. All plates were incubated for 24–48 h at 30 °C. After incubation, the colonies were purified, subcultured, and stored at 4 °C for further analysis.

#### Endophytic bacteria

One-gram algae of each sample were washed gently 3 to 4 times in sterile seawater, followed by a two-minute wash in 70% ethanol and in 2% sodium hypochlorite for one minute. The samples were then rinsed with sterile seawater for five minutes with vigorous shaking and dried with sterile filter paper [17]. The algal samples were cut into sections about 2 to 3 cm long by a sterilized knife or scalpel. These sections were then inoculated into nutrient broth with serial dilution series, and then inoculated on ordinary nutrient medium plates with seawater at 37 °C for 24 h to allow the growth of associated endophytes.

### Enumeration of isolated epiphytic and endophytic bacteria

Plates that were inoculated by epiphytes and endophytes bacteria were enumerated using the serial dilution agar plating method. The plates were incubated at 30 °C till growth was observed. The results were expressed as colony-forming units (CFU/cm^2^ algae) using the following equation [18].

Bacterial count (CFU/cm^2^) = Number of bacterial colonies (CFU/ml) / (Volume plated (ml) x total dilution used).

### Statistical analysis of bacterial enumeration

All the experiments were carried out in triplicate. Results are expressed as mean ± standard deviation. The two-way analysis of variance (ANOVA) was performed to find the effect of the algal morphotypes and spatial variation on the epiphytic and endophytic bacterial counts. The macroalgal thalli were subdivided into six groups according to the thalli morphology and surface characteristics [19]. The first group is the branched thalli (*Cladophora pellucida*). The second group is coarsely branched (*Codium* sp., *Gelidium crinale*, and *Pterocladiella capillacea*). The third group is the jointed calcareous (*Corallina mediterranea*, *Corallina officinalis*, and *Jania rubens*). The fourth group is thick leathery (*Petalonia fascia*). The fifth group is the sheet-like thalli (*Ulva lactuca*), while the sixth group is the filamentous *Ulva intestinalis*. A one-way analysis of variance (ANOVA) was used to investigate the relation between bacterial enumeration and the distribution of algae associated with, according to the sites located on marine shores. Statistical analysis was performed using Minitab Statistical Software. We used the analysis of variance (ANOVA) and the least significant difference (LSD) test to compare the study’s parameters, which included counting the number of isolated bacterial cells that were linked to different types of macroalgae and their distribution at different sites along the shoreline. The mean differences greater than LSD were considered significant at the 0.05 level.

$$\:{\text{L}.\text{S}.\text{D}}_{0.05}={\text{t}}_{(\text{dfw},\frac{{\upalpha\:}}{2})}\sqrt{\text{MSe*}(\frac{1}{\text{n}}+\frac{1}{\text{n}`})}$$

Where, t is the tabulated t value at the 0.05 level of probability and the degree of freedom is 17 or 16, MSe is the mean square of error, and n is the number of replicates.

### Phenotypic and physiological characterization of bacteria associated with macroalgae

Phenotypic and physiological characterizations of the algae-associated bacteria were carried out according to Bergey’s Manual of Systemic Bacteriology [20] and Akulava et al. [21]. Bacterial isolates were cultured on nutrient agar (NA) plates at different temperatures (3, 30, and 50 ℃), NaCl concentrations (0, 5, 10, and 15%), pH levels (5, 7, and 9), and with or without seawater. Different enzyme (amylase, casein, alginase, catalase, and cellulase) activities were also determined for the isolated bacteria. We subcultured the sixty-five isolates in nutrient broth and incubated them at 37 °C for 24 h. The inoculated nutrient agar plates contain different substrates such as starch, casein, alginate, and cellulose. Only isolates that are capable of using the substrate as a carbon source will yield positive results. Carbon sources play an important role in enzyme production, and different bacteria utilized different C sources for their growth and metabolism. So, the media would identify isolates that are capable of producing the specific enzyme for each substrate. After incubation at 37 °C for 24 h, the plates were examined, and the enzyme activity for each substrate was determined based on the visual observation of the clear zone formed by the bacterial colony [22].

## Results

### Classification and geographical abundance of the collected algal species

**Fig. 2:**
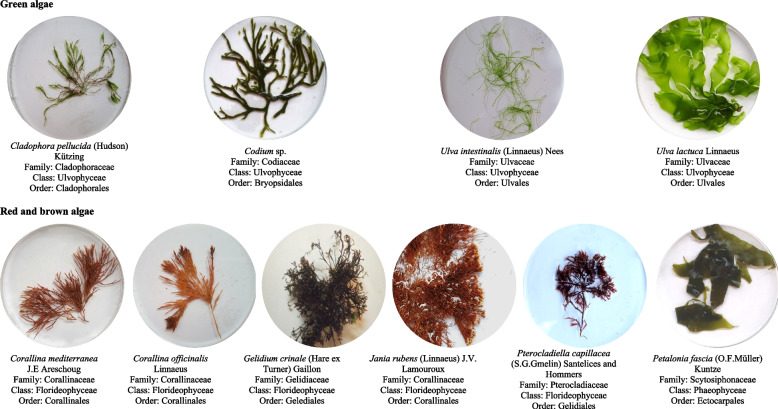
Collected algal samples from Alexandria shores (Abu Qir, Montaza, Miami and the Eastern Harbor), Egyptian Mediterranean Sea during winter and their taxonomic positions (All photos were taken by Samia S. Abouelkheir in the Marine Microbiology Laboratory, National Institute of Oceanography and Fisheries (NIOF), Alexandria, Egypt)

**Fig. 3:**
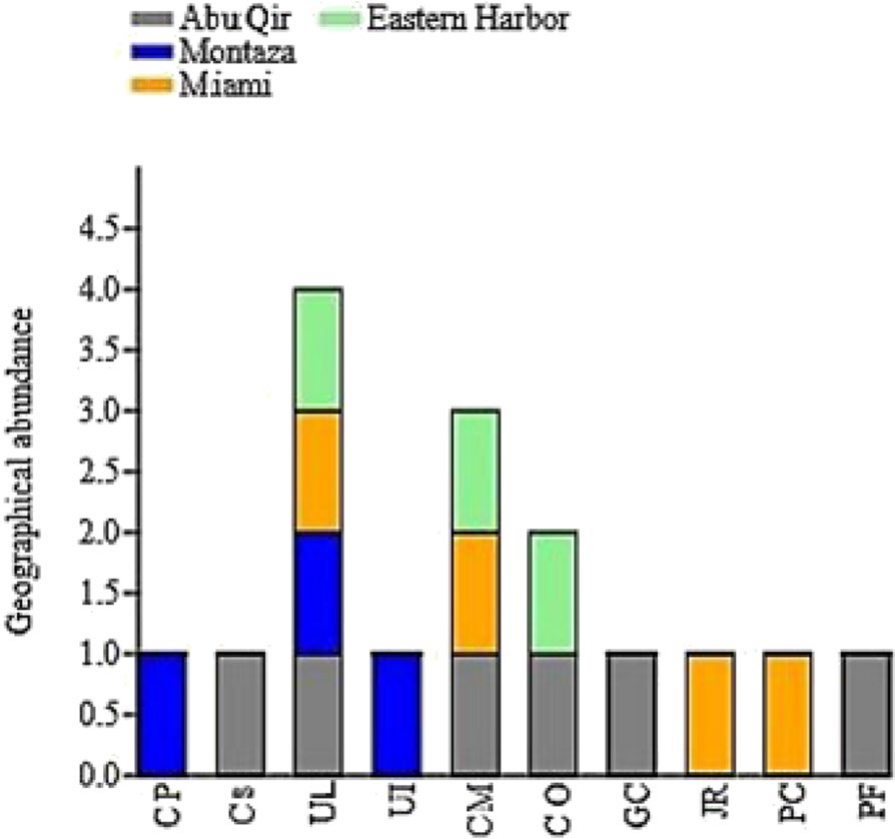
Stacked chart (Past3.exe program) of geographical abundance and distribution of marine macroalgae species in the four sites

Ten species were collected from the four sites, belonging to three taxonomical classes. The class Chlorophyceae (green algae) was represented by *Cladophora pellucida*, *Codium* sp., *Ulva lactuca*, and *Ulva intestinalis*. The class Rhodophyceae (red algae) was represented by *Corallina mediterranea*, *Corallina officinalis*, *Gelidium crinale*, *Jania rubens*, and *Pterocladiella capillacea*, while the Phaeophyceae (brown algae) was only represented by *Petalonia fascia* (Fig. 2). The geographical abundance and distribution of the ten marine macroalgal species are shown in Table 1; Fig. 3.

**Table 1:** The distribution of the ten marine macroalgal species

Macroalgae	Abu Qir	Montaza	Miami	Eastern Harbor
*Cladophora pellucida* (CP)		+		
*Codium *sp. (CS)	+			
*Ulva lactuca* (UL)	+	+	+	+
*Ulva intestinalis* (UI)		+		
*Corallina mediterranea* (CM)	+		+	+
*Corallina officinalis* (CO)	+			
*Gelidium crinale* (GC)	+			
*Jania rubens* (JR)			+	
*Pterocladiella capillacea* (PC)			+	
*Petalonia fascia* (PF)	+			

### Enumeration of bacteria associated with macroalgae (epiphytes and endophytes)

The results of the two-way ANOVA test revealed that the algal morphotypes and spatial variation had neither effect on the count of epiphytic bacteria (*P* = 0.42 for algal morphotypes, *P* = 0.18 for sites) nor the endophytic ones (*P* = 0.92 for algal morphotypes, *P* = 0.86 for sites) (Table 2). Therefore, the counting of bacteria associated with marine macroalgae was carried out using either nutrient agar medium only (NA) or supplemented with algal extract (NA_AE). Data in Table 3 represent epiphytic and endophytic bacterial average counts of 3 replicates in the 4 sites. Statistical analysis in Table 3 showed values with the same or different capital letters; similar letters mean that there is no significant difference at the LSD 0.05 level of probability.

**Table 2:** Two-way analysis of variance (ANOVA) results between the algal morphotypes and spatial variation revealed no influence on the epiphytic and endophytic bacterial counts (CFU)

**Epiphytic bacteria**						
ANOVA						
*Source of Variation*	*SS*	*df*	*MS*	*F*	*P-value*	*F crit*
Rows (morphotypes)	1783525000	9	198169444.4	**1.06**	**0.42**	2.25
Columns (sites)	988675000	3	329558333.3	**1.77**	**0.18**	2.96
Error	5025575000	27	186132407.4			
Total	7797775000	39				
**Endophytic bacteria**						
ANOVA						
*Source of Variation*	*SS*	*df*	*MS*	*F*	*P-value*	*F crit*
Rows (morphotypes)	538749000	9	59861000	**0.41**	**0.92**	2.25
Columns (sites)	112371000	3	37457000	**0.25**	**0.86**	2.96
Error	3980719000	27	147434037			
Total	4631839000	39

**Table 3:** Estimated bacterial count (CFU) of the macroalgae collected from the four Alexandria Mediterranean Sea sites grown on nutrient agar medium (NA) only and medium containing algal extract (NA_AE)

**Station**	**Epiphytic Bacteria**		**Endophytic Bacteria**	
	**NA**	**NA_AE**	**NA**	**NA_AE**
Site 1 (Abu Qir)	26.3 ^(A)^	31.8 ^(A)^	15.4 ^(A)^	56.8 ^**(AB)**^
Site 2 (Montaza)	6.6 ^(B)^	43.0 ^**(AB)**^	16.3 ^(A)^	34.8 ^**(B)**^
Site 3 (Miami)	25.3 ^(A)^	26.3^(A)^	11.9 ^(A)^	27.5 ^**(B)**^
Site 4 (Eastern Harbor)	15.0 ^(A)^	41.0 ^**(AB)**^	19.0 ^(A)^	34.7 ^**(B)**^

^*^Means followed by the same capital letters are not significantly different at LSD 0.05 level of probability

The results of the one-way ANOVA test showed that adding algal extract to the culture medium has a positive effect on improving the vital activity of the bacterial isolates and helping them to grow. The effect of the supplemented algal extract fluctuated depending on the isolates at each site. In Abu Qir site, the algal extract strongly affected the growth of the endophytic bacteria by increasing their numbers by 3.8 times, while there was no significant effect on the epiphytic one. Moreover, the addition of algal extract caused a significant increase in both epiphytes and endophytes in Montaza site by 7 and 2 times, respectively. However, algal extract made no significant effect on all epiphytic isolates in Miami and showed a significant increase in the count of endophytes by 1.8 times. Eastern Harbor showed a significant effect on epiphytic bacteria growth by 2.7 times when it’s supplemented with algal extract; it also affected endophytic bacteria growth by 1.8 times compared to the medium alone.

### Enumeration of bacterial communities associated with green, red, and brown classes of macroalgae

**Fig. 4:**
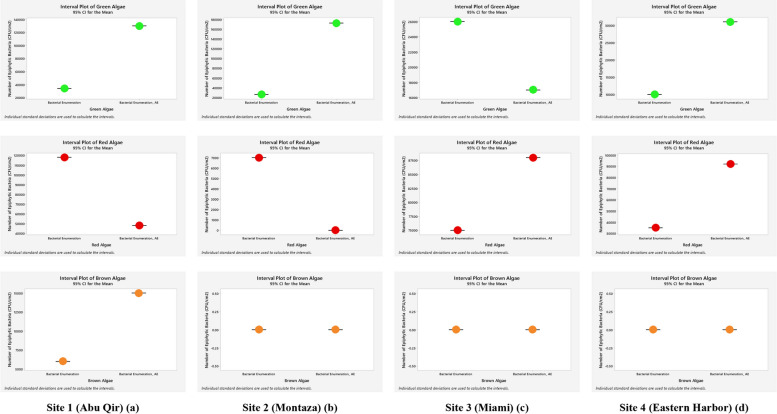
Interval plot (Minitab 19 program) of estimated epiphytic bacterial count (CFU/cm^2^) associated macroalgae (Green, Red, and Brown) collected from the four Alexandria Mediterranean Sea sites grown on nutrient agar medium (NA) only and medium containing algal extract (NA_AE)

**Fig. 5:**
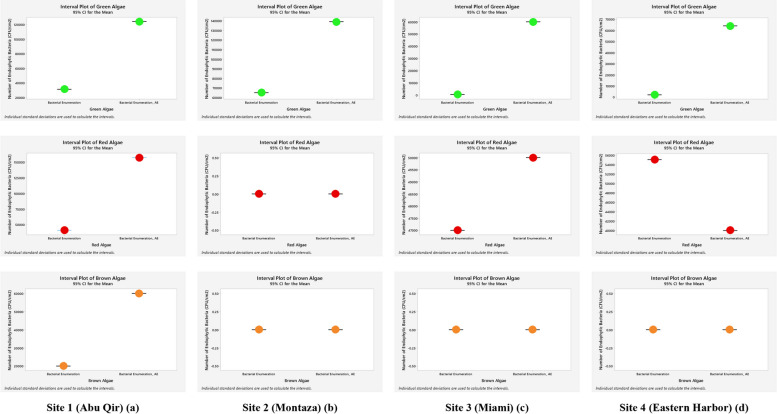
Interval plot (Minitab 19 program) of estimated endophytic bacterial count (CFU/cm^2^) associated macroalgae (Green, Red, and Brown) collected from the four Alexandria Mediterranean Sea sites grown on nutrient agar medium (NA) only and medium containing algal extract (NA_AE)

Based on the comparison by relative abundance of bacteria associated macroalgae using the interval plot with confidence interval of 95% of descriptive statistics analyses (Fig. 4), most of the epiphytic bacteria, linked with green algae after supplementing the media with algal extract, were increased to all sites except for Miami. However, the relative abundances of epiphytic bacteria on red algae differed; they were decreased in Abu Qir and Montaza sites while they increased in both Miami and Eastern Harbor ones. Epiphytic bacteria-associated brown alga were prevalent and grew only in Abu Qir site, and were unaffected by algal extract. The overall conclusion from this figure is that the epiphytic-associated bacteria in almost all sites are dependent on supplementing the media with algal extract. The results shown in Fig. 5 confirmed that the endophytic bacteria associated with green algae increased in all sites. While those associated with red algae were increased only in Abu Qir and Miami sites and decreased in Estern Harbor, whereas in Montaza the red algae were not represented. Finally, endophytic bacteria associated with the brown alga showed enrichment only in Abu Qir site, with no effect on Montaza, Miami, or Eastern Harbor, respectively.

### Enumeration of bacterial communities associated with the ten recorded species of macroalgae

**Fig. 6:**
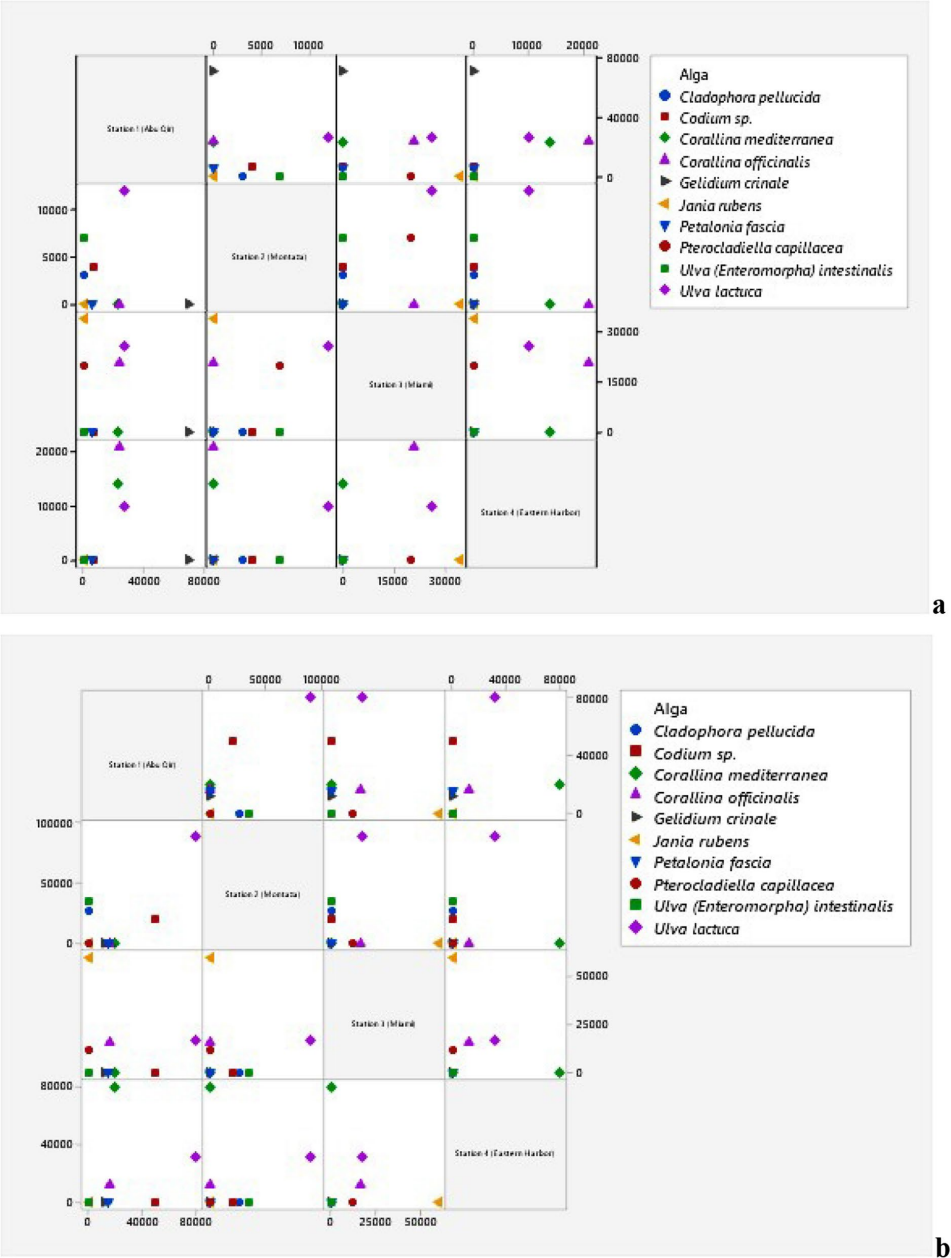
Matrix Plot (Minitab 19 program) of epiphytic bacteria (**a**) and after algal extract addition (**b**) on different macroalgal species alongside the four sites

The addition of algal extract to the enrichment media altered the bacterial numbers associated with each of the ten macroalgae. However, the number of bacteria was different when algal extracts were present compared to when they were not. The epiphytic bacterial number estimated for *Cladophora pellucida* found only at Montaza increased ninefold upon the addition of algal extract (Fig. 6a, b). The number of bacteria colonized *Codium* sp. increased after the addition of the algal extract in both Abu Qir and Montaza sites by sevenfold and fivefold, respectively. Similarly, the number of bacteria found in *Ulva intestinalis* found only at Montaza site rose five times when the algal extract was added. The number of bacteria found on *Ulva lactuca* in Abu Qir, Montaza, and Eastern Harbor was 2.96, 7.5, and 3.1 times higher than when algal extract wasn’t added while, the number of bacteria found at the Miami site went down by 1.5 times. For *Corallina mediterranea*, the number of bacteria decreased by 1.15 fold in Abu Qir site and then increased by 5.7 fold in the Eastern Harbor. In the case of *Corallina officinalis*, the number of bacteria is totally affected by the addition of algal extract and decreased in the three sites (Abu Qir, Miami, and Eastern Harbor by 1.5, 1.31, and 1.8 fold, respectively). The number of epiphytic bacteria associated with *Gelidium crinale*, which appeared only in Abu Qir site, decreased by 5.9 fold while increasing by 2.5 fold with *Petalonia fascia*, which also appeared only at the same site. Although the epiphytic bacteria associated with *Jania rubens* increased by 1.76 fold in Miami site, the associated bacteria with *Pterocladiella capillacea* decreased in number in Miami site by 1.7 fold while totally disappearing in Montaza site.

**Fig. 7:**
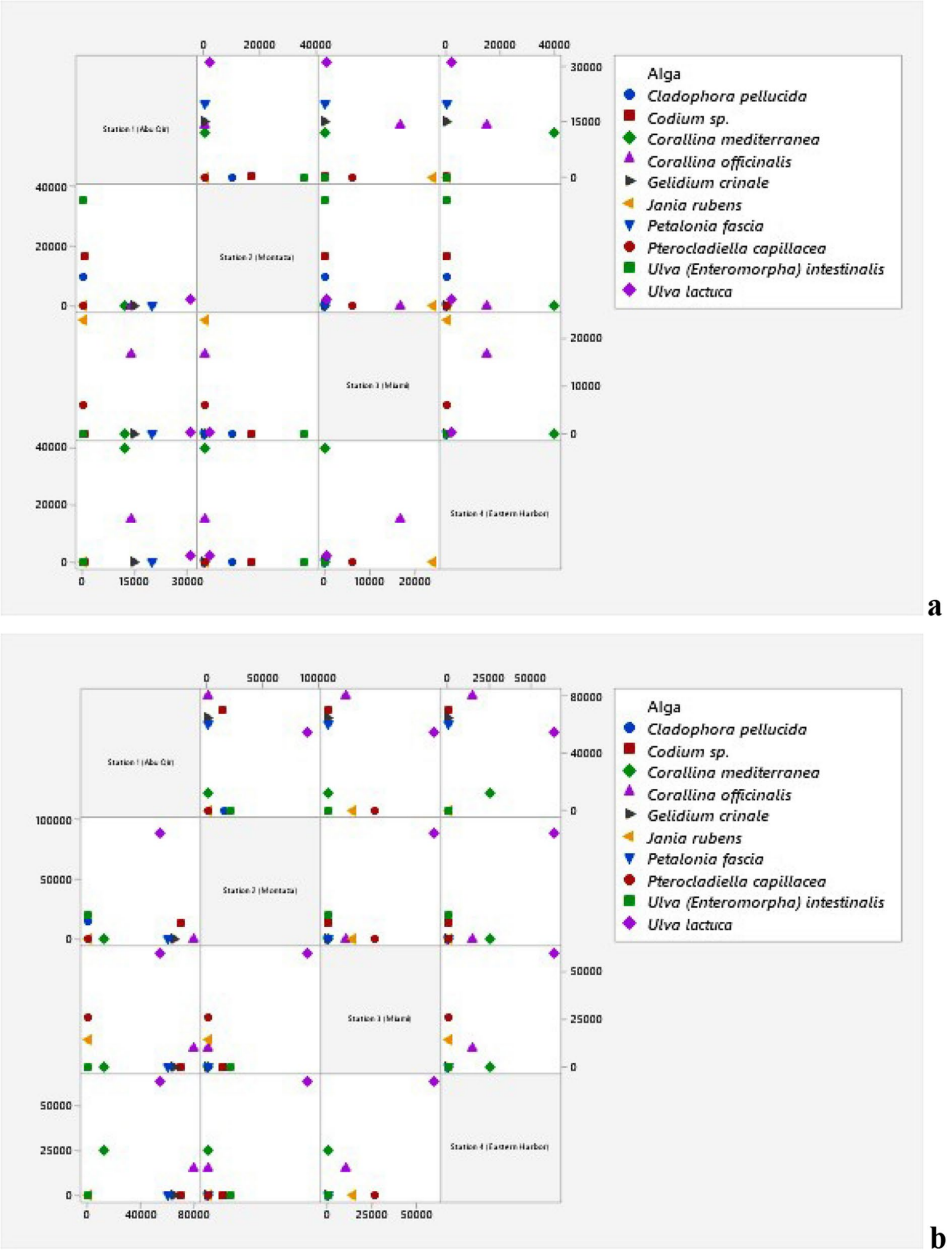
Matrix Plot (Minitab 19 program) of endophytic bacteria (**a**) and after algal extract addition (**b**) on different macroalgal species alongside the four sites

The changes in the number of endophytic bacteria were also different between the collected various macroalgae. On the diverse macroalgae, the number of bacteria was not identical and was affected by the addition of algal extracts (Fig. 7a, b). The endophytic bacteria associated with *Cladophora pellucida*, *Ulva lactuca*,* Gelidium crinale*,* Pterocladiella capillacea*, and *Petalonia fascia* increased while decreasing with *Ulva intestinalis* and *Jania rubens*.

Notably, after the addition of algal extract, the number of presented endophytic bacteria associated with *Codium* sp. and *Corallina officinalis* increased in Abu Qir while it decreased with *Codium* sp. and *Corallina mediterranea* in Montaza and in Eastern Harbor with *Corallina mediterranea*. Moreover, the number of bacteria present on both corallinates, *Corallina mediterranea* and *Corallina officinalis*, in Abu Qir and Eastern Harbor sites, respectively, remained the same and unchanged.

### Culture-dependent isolation of marine algal epiphytes and endophytes

**Fig. 8:**
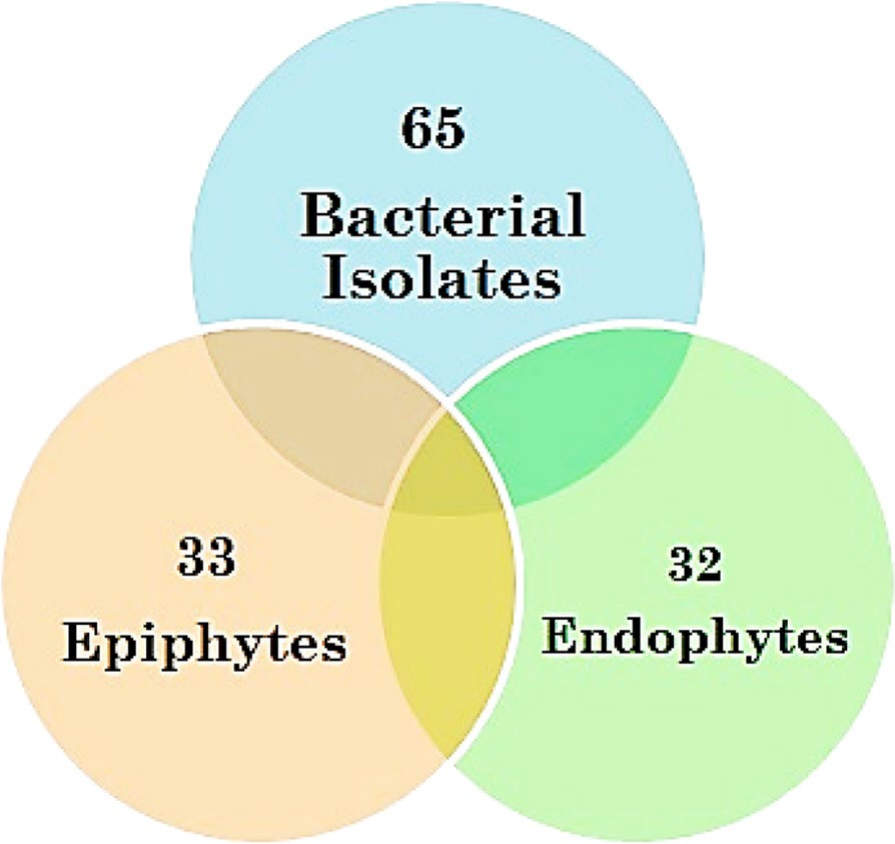
Venn shape (Microsoft Excel 2010 software program) of bacterial isolates showing the numbers of isolated epiphytic and endophytic bacteria

A total of sixty-five bacterial isolates of culturable aerobic bacteria were isolated from ten marine macroalgae collected from four sites in the Alexandria Mediterranean Sea. The results confirmed that thirty-three isolates were epiphytes and thirty-two were endophytes (Fig. 8).

The majority of epiphytic and endophytic bacteria were isolated from the thallus of *Ulva lactuca*, around 17 isolates, followed by *Corallina mediterranea* (9 isolates), *Corallina officinalis* (7 isolates), *Petalonia fascia*, *Ulva intestinalis*, *Pterocladiella capillacea* (5 isolates), *Codium* sp., *Gelidium crinale*,* Cladophora pellucida*, and *Jania rubens* (4 isolates) (Table 4). All the isolates showed growth after 24 h of incubation at 30 °C. The isolates varied in appearance from one another in terms of colour, shape, and other phenotypic traits.

**Table 4:** List of epiphytic and endophytic bacterial isolates obtained from different macroalgae distributed in the four stations

**Stations**	**Abu Qir (A)**		**Montaza (M)**		**Miami (Y)**		**Eastern Harbor (E)**	
**Bacteria**								
**Epiphytic (E)**	**Algae Name**	**Isolate Code**	**Algae Name**	**Isolate Code**	**Algae Name**	**Isolate Code**	**Algae Name**	**Isolate Code**
	(**CM**)		(**CP**)					
	*Corallina mediterranea* 1 *Corallina mediterranea* 2 *Corallina mediterranea* 3	CMEA1 CMEA2 CMEA3	*Cladophora pellucida* 1 *Cladophora pellucida* 2	CPEM1 CPEM2			*Corallina mediterranea*	CMEE
	(**CO**)		(**UI**)		*Corallina officinalis*	COEY	*Corallina officinalis*	COEE
	*Corallina officinalis* 1 *Corallina officinalis* 2	COEA1 COEA2	*Ulva intestinalis* 1 *Ulva intestinalis* 2	UIEM1 UIEM2				
	(**CS**)				(**JR**)			
	*Codium* sp. 1 *Codium* sp. 2 *Codium* sp. 3	CSEA1 CSEA2 CSEA3			*Jania rubens* 1 *Jania rubens* 2	JREY1 JREY2		
	(**GC**)							
	*Gelidium crinale* 1 *Gelidium crinale* 2	GCEA1 GCEA2						
	(**UL**)		*Ulva lactuca* 1 *Ulva lactuca* 2 *Ulva lactuca* 3	ULEM1 ULEM2 ULEM3	*Ulva lactuca* 1 *Ulva lactuca* 2	ULEY1 ULEY2	*Ulva lactuca* 1 *Ulva lactuca* 2	ULEE1 ULEE2
	*Ulva lactuca* 1 *Ulva lactuca* 2	ULEA1 ULEA2						
	(**PF**)				(**PC**)			
	*Petalonia fascia* 1 *Petalonia fascia* 2 *Petalonia fascia* 3	PFEA1 PFEA2 PFEA3			*Pterocladiella capillacea* 1 *Pterocladiella capillacea* 2	PCEY1 PCEY2		
**Endophytic (N)**								
	*Corallina mediterranea* 1	CMNA1	*Cladophora pellucida* 1 *Cladophora pellucida* 2	CPNM1 CPNM2	*Corallina officinalis*	CONY	*Corallina officinalis*	CONE
	*Corallina officinalis* 1 *Corallina officinalis* 2	CONA1 CONA2			*Jania rubens* 1 *Jania rubens* 2	JRNY1 JRNY2	*Corallina mediterranea* 1 *Corallina mediterranea* 2 *Corallina mediterranea* 3 *Corallina mediterranea* 4	CMNE1 CMNE2 CMNE3 CMNE4
	*Codium* sp. 1	CSNA1	*Ulva intestinalis* 1 *Ulva intestinalis* 2 *Ulva intestinalis* 3	UINM1 UINM2 UINM3	*Pterocladiella capillacea* 1 *Pterocladiella capillacea* 2 *Pterocladiella capillacea* 3	PCNY1 PCNY2 PCNY3		
	*Gelidium crinale* 1 *Gelidium crinale* 2	GCNA1 GCNA2						
	*Ulva lactuca* 1 *Ulva lactuca* 2	ULNA1 ULNA2	*Ulva lactuca* 1 *Ulva lactuca* 2 *Ulva lactuca* 3	ULNM1 ULNM2 ULNM3	*Ulva lactuca* 1 *Ulva lactuca* 2	ULNY1 ULNY2	*Ulva lactuca* 1	ULNE1
	*Petalonia fascia* 1 *Petalonia fascia* 2	PFNA1 PFNA2						

### Phenotypic and physiological characterization of bacteria associated with macroalgae

All sixty-five cultivable epiphytic and endophytic bacterial isolates obtained from the thallus of the ten different macroalgae were plated on nutrient agar. The phenotypic examination of the obtained isolates showed differences in colony shape, colour, and mucoid after being grown on nutrient agar plates after 24 h of incubation at 30 ± 2 °C. Colours ranged from white to pale orange. The colonies showed differences as well in cell viscosity, where some of the colonies were mucoid and the others were normal.

**Fig. 9:**
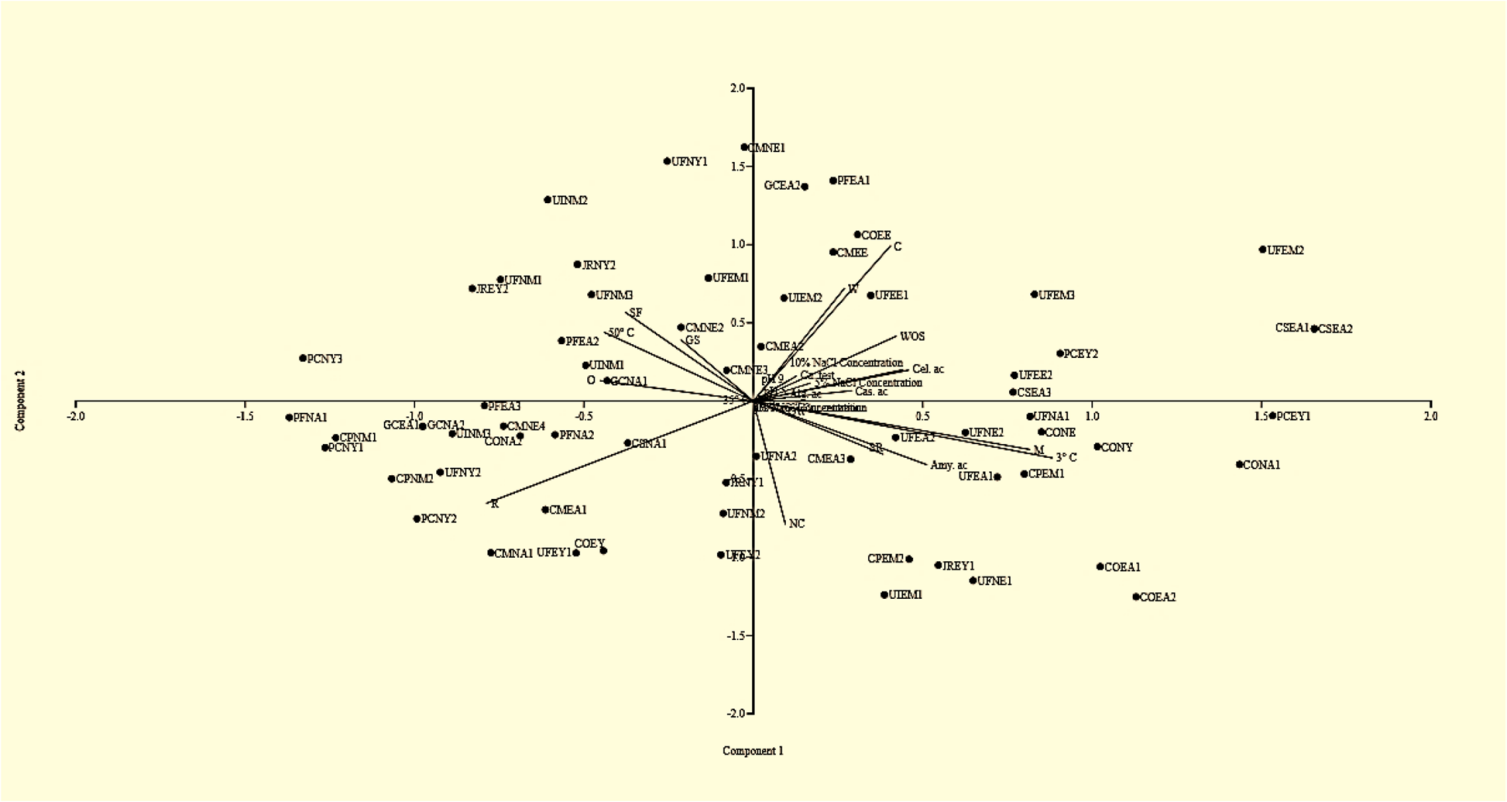
Principal components analysis (Past3.exe program) of the fifty-six isolates identifying the similar potential physiological factors and phenotypic properties influencing the bacterial communities associated with macroalgae

Correlation analysis among prokaryotic communities and physicochemical properties was accomplished by principal components analysis (Fig. 9). According to the results, all physicochemical properties, including temperature, salinity, pH, and their ability to grow with or without seawater, were key factors that affected the prokaryotic community and significantly correlated with these physicochemical factors with *P* ≈ 0.05. The components analysis of the sixty-five isolates presented in Fig. 9 showed that colony colour varies from white (W), off-white (OW), non-coloured (NC), and coloured orange (O). 50% of the isolates were mucoid (M), and the others were non-mucoid (NM). Microscopic examination of isolates showed differences in cell shapes; they varied between rod (R), short rod (SR), and cocci (C). Gram stain (GS) discriminates between Gram-positives and negatives; most epiphytes and endophytes associated with green, red, or brown algae were Gram-positive. All bacterial isolates associated with the brown alga were non-spore-forming, while only endophytes isolated from green algae were spore-formers (SF). Major epiphytes were non-spore-formers. The majority of epiphytes and endophytes isolated from red algae were non-spore-formers.

The physiological properties of the studied isolates showed their ability to grow with (WS) or without seawater (WOS) as a vital factor for their growth. Most of the bacterial isolates need seawater as an essential element for their growth, but a few isolates showed growth without seawater. The isolates showed growth at 0% and 5% NaCl concentrations, and as the salt concentration increased (10%), the growth decreased until at 15% NaCl there was no growth noticed. The optimum temperature degree for the isolate’s growth was 30 °C. Although the neutral medium (pH 7) was the best for isolate growth, isolates also grew at pH 5 and 9.

Among these isolates, the majority of them showed wide range of enzyme activities (Ac) (Table 5). All epiphytic and endophytic bacteria isolated from Abu Qir site and cultivated at 30℃ showed no catalase (Cat) production but showed the activity of cellulase (Cel), alginase (Alg), and caseinase (Cas). Most of these isolates had amylase (Amy) activity. Bacterial isolates from the Eastern Harbor site showed no catalase activity except one epiphyte isolate (COEE) and no amylase activity for all isolates. In addition, all bacterial isolates from Montaza and Miami showed enzymes activities for cellulase, alginase, caseinase and amylase, but has no catalase activity except two epiphyte isolates from Montaza site. From the sixty five isolates, 26% of the isolates were with amylolytic activity and 40% of the isolates were with cellulolytic activity.

**Table 5:** Shows the results of a qualitative test on the enzyme activities of epiphytic and endophytic bacterial isolates

**Stations**	**Abu Qir (A)**					**Montaza (M)**					**Miami (Y)**					**Eastern Harbor (E)**				
**Epiphytic Bacteria (E)**	**Isolate** **Code**	**Amyl-ase**	**Casei-nase**	**Algin-ase**	**Cellul-ase**	**Isolate Code**	**Amyl** **-ase**	**Casei-nase**	**Algin-ase**	**Cellul-ase**	**Isolate Code**	**Amyl** **-ase**	**Casei-nase**	**Algin-ase**	**Cellul-ase**	**Isolate Code**	**Amyl** **-ase**	**Casei-nase**	**Algin-ase**	**Cellul** **-ase**
	CMEA1	**-**	**-**	**-**	**-**	CPEM1	** + + **	**-**	**-**	**-**						CMEE	**-**	**-**	**-**	**-**
	CMEA2	**-**	** + + **	**-**	**-**	CPEM2	**-**	**-**	**-**	**-**										
	CMEA3	** + **	**-**	**-**	**-**															
	COEA1	** + **	**-**	**-**	** + **	UIEM1	**-**	** + + **	**-**	** + **	COEY	-	-	-	-	COEE	**-**	**-**	** + + **	**-**
	COEA2	** + + **	**-**	**-**	** + **	UIEM2	**-**	**-**	**-**	**-**										
	CSEA1	** + **	**-**	**-**	** + + **						JREY1	+ +	-	-	+					
	CSEA2	** + **	**-**	**-**	** + **						JREY2	-	-	-	-					
	CSEA3	**-**	**-**	**-**	** + **															
	GCEA1	**-**	**-**	**-**	**-**															
	GCEA2	**-**	** + **	**-**	**-**															
	ULEA1	** + + + **	** + + **	**-**	**-**	ULEM1	**-**	**-**	** + **	**-**	ULEY1	+ +	-	-	-	ULEE1	**-**	**-**	** + **	**-**
						ULEM2	**-**	** + **	**-**	** + **	ULEY2	+	-	-	-	ULEE2	**-**	**-**	**-**	**-**
	ULEA2	**-**	** + + **	**-**	** + + **	ULEM3	**-**	**-**	**-**	**-**										
	PFEA1	**-**	**-**	**-**	** + + **						PCEY1	+ +	-	+	+					
	PFEA2	**-**	**-**	**-**	** + **						PCEY2	-	+ + +	-	-					
	PFEA3	**-**	**-**	**-**	**-**															
**Endophytic Bacteria (N)**																				
	CMNA1	**-**	**-**	**-**	**-**	CPNM1	**-**	**-**	**-**	**-**	CONY	+ +	+ +	+	-	CONE	**-**	**-**	** + + **	** + **
	CONA1	** + **	**-**	**-**	** + + **	CPNM2	**-**	**-**	**-**	**-**	JRNY1	-	-	-	-	CMNE1	**-**	** + **	**-**	** + + **
	CONA2	**-**	**-**	**-**	**-**						JRNY2	-	-	-	+	CMNE2	**-**	**-**	**-**	**-**
	CSNA1	**-**	**-**	** + + **	**-**	UINM1	** + + + + **	**-**	**-**	** + + **	PCNY1	-	-	-		CMNE3	**-**	**-**	**-**	**-**
	GCNA1	**-**	** + **	**-**	**-**	UINM2	**-**	**-**	**-**	**-**	PCNY2	-	-	-	-	CMNE4	**-**	**-**	**-**	** + **
	GCNA2	**-**	**-**	**-**	**-**	UINM3	** + + + + **	**-**	**-**	** + **	PCNY3	-	-	-	+					
	ULNA1	** + **	** + **	**-**	** + **	ULNM1	**-**	**-**	**-**	**-**	ULNY1	-	-	-	+	ULNE1	**-**	**-**	**-**	**-**
	ULNA2	** + **	** + **	**-**	**-**	ULNM2	**-**	** + + **	**-**	**-**	ULNY2	-	-	-	+					
	PFNA1	**-**	**-**	**-**	**-**	ULNM3	**-**	**-**	**-**	**-**										
	PFNA2	**-**	**-**	**-**	**-**															

## Discussion

Marine algae are photosynthetic organisms living in the seas and oceans. In recent decades, much research has focused on seaweed-associated bacterial communities to understand their structure, succession, and dynamics in connection to bacterial-seaweed interactions and ecology. In addition, comprehensive studies of total algal surface bacterial populations are rare. Therefore, the present study focuses on studying the relationships between marine macroalgae and their associated bacteria at four different sites: Abu Qir, Montaza, Miami, and Eastern Harbor. The bacteria associated with macroalgae were highly diversified in the study area, indicating the richness of associated communities and the important role of the host algae, which offer a highly suitable natural substratum to these organisms, similarly to what was previously reported by Shams El-Din et al. [23].

However, as clearly observed from Figs. 2 and 3, the morphotypes of algal hosts and their geographical distribution were more effective for the abundance of bacterial epiphytes and endophytes from one study site to another. The recorded algae have different shapes and looks, such as *Ulva intestinalis* having narrow-bladed filamentous thallus, *Ulva lactuca* having thin, sheet-like fronds [24], and *Cladophora pellucida* having a branching thallus [25]. *Corallina mediterranea* and *Corallina officinalis* thalli are dichotomously branching jointed calcareous [24], but *Codium* sp. is coarsely branched looked like a spongy alga joined by flat discs [26]. *Pterocladiella capillacea* thalli has alternating branches [27], but *Jania rubens* is a jointed calcareous and branching thallus [28]. The thallus of *Petalonia fascia* was flattened thick leathery [29], while *Gelidium crinale* was made up of cartilaginous coarsely branched thalli [30]. Algal thalli may represent an order of magnitude greater surface area for bacterial colonization and growth. Bacterial flora composition can change over thallus parts due to biotic and abiotic factors [31]. In addition, different algal species support different bacterial communities in the same habitat [32].

Notably, the other factors that affect the occurrence of macroalgae and their associated bacteria are environmental conditions such as temperature, pH, salinity, and nutrients as confirmed by our principle component analysis. The discharge of wastewater from the nearby urbanized area had an impact on Abu Qir Bay’s seawater salinity, which ranged from 37.24 to 38.21 during the winter (16.10 to 17.16 °C) season [33]. The water’s pH ranged between 7.13 and 8.61, suggesting a slightly alkaline nature due to phytoplankton and photosynthesis processes. These characteristics were obviously the reason why we collected six species from the Abu Qir site (*Codium* sp., *Ulva lactuca*, *Corallina mediterranea*, C. *officinalis*,* Gelidium crinale*, and *Petalonia fascia)*, followed by Miami recording four species from ten (*Ulva lactuca*, *Corallina mediterranea*, *Jania rubens*, and *Pterocladiella capillacea*). Miami seawater salinities ranged from 38.4 to 39.7‰, with no industrial activity on the coast. Domestic waste contributes, avoiding heavy metals and making it a suitable site for algal growth [28]. Both sites, Montaza and Eastern Harbor, harboured three macroalgal species. Only the Montaza site harboured *Cladophora pellucida*, *Ulva lactuca*, and *Ulva intestinalis*. Montaza seawater pH value also ranged between 7.13 and 8.61, while the salinity showed variations between 37.98 and 38.57‰ [34]. *Ulva lactuca*, *Corallina mediterranea*, and *C. officinalis* were found at Eastern Harbor. The pH of Eastern Harbor water fluctuated between 7.43 and 8.21 in 2012, indicating the aquatic ecosystem’s redox potential and productivity level. A variety of *Ulva* spp. was found at more than one site in our study area. *Ulva intestinalis* was collected only from the Montaza site, while *Ulva lactuca* was found at all four sites. *Ulva* is well known for its wide distribution in marine, freshwater, and brackish environments on a global scale in the intertidal and shallow subtidal zones of rocky shores all over the world [35].

Because seaweed surfaces are rich in nutrients and provide protection, opportunistic bacteria are found anywhere there is organic material. Generally speaking, marine macroalgae are linked to certain bacterial communities that are quite different from those found in the nearby saltwater [36]. It is well Known that a variety of bacterial symbionts live on the surfaces of seaweeds, aiding in the development of their morphology and defense systems [37]. However, awareness of the variety of epiphytic bacteria and their intricate relationships with their hosts remains limited. For instance, a vast range of maritime environments and an exceptionally rich and diversified seaweed flora may be found in Southern Africa [7]. In several locations over the last few decades, researchers have looked into seaweed-associated epiphytic bacterial communities in great detail using culture-dependent investigations and community fingerprinting evaluations [38, 39].

In reality, in the present study, there is no significant difference between the counts of epiphytic and endophytic bacteria from the point of view of algal morphotypes and different sites. Thus, the host didn’t play a significant role in the bacterial count, which was confirmed by the two-way ANOVA test. These findings disagree with those previously published by Shams El-Din et al., who reported a significant correlation between the total count of epiphytic microalgae and the morphotypes of hosts, giving the order of preference first to the branched thalli, followed by the smooth surface, and then the mucilaginous one [23]. Furthermore, this may be due to the algal host’s increased surface area in this direction [23].

However, as Table 3; Figs. 4 and 5 demonstrate readily evident, the statistical findings of the current investigation showed considerable differences in the epiphytic and endophytic total count with the addition of algal extracts. This might be because some interactions between seaweeds and the bacteria they are connected with, can be triggered by the components of the algal cell walls and secondary metabolites which differes from one algal group to another [40]. Bacteria reside not just on the surfaces of algae but also inside their tissues [36]. Furthermore, with the addition of algal extracts, much differences were discovered at the Abu Qir and Miami sites for the overall count of the only epiphytic bacteria (Table 3).

The addition of algal extracts had a negative correlation with the total number of epiphytic bacteria on the green algae at the Miami site and a negative impact on the number of epiphytic bacteria on the red algae at both the Abu Qir and Miami sites (Fig. 4). The entire number of endophytic bacteria on the red algae and the addition of algal extracts at the Eastern Harbor site only demonstrated this negative relationship (Fig. 5). This implies that the growth may be being inhibited by other reasons, such as the release of hazardous compounds. According to Peckol & Putnam [41], this tendency indicates that herbivores have different grazing preferences. This indicates that the distribution and impact of these animals are affected by the poisonous exudate that *U. lactuca* releases. Cressey and his colleagues corroborated our findings [42]. According to reports, there are potential health risks associated with seaweed because of naturally existing elements like iodine and the bioaccumulation of potentially harmful chemicals including arsenic, lead, cadmium, and mercury. If bacteria or viruses are found in the marine environment, they may infect seaweed. Furthermore, there is some indication that marine biotoxins may also infect seaweed, although this is not confirmed. These will be process-specific characteristics rather than probable seaweed-specific ones [36].

Besides biosynthesis and metabolism, a subtle difference in functional genes responsible for bacterial chemotaxis was observed, with such genes being relatively high in red seaweeds compared to green and brown seaweeds. In general, some types of bacteria make regulatory chemicals that look like cytokinins. These chemicals may help restore seaweed to its normal shape if it gets damaged by the tides [43]. However, the complexity of microbial systems, coupled with the low concentrations of these molecules in water and their rapid uptake, are still challenges, making it difficult to gain an in-depth understanding of algal surface microhabitats and oceanic functioning [7]. Several studies have shown that the diversity of endophytes living on algae varies depending on the genus of the algae [44, 45]. Changes in the algae’s environment, such as temperature and nutrient levels, will affect the variety of endophytes living on it. Thus, different endophytes were identified from the same host (*Ulva* sp.) that was collected at three locations [46].

*Ulva lactuca* harbored a relatively higher total count of epiphytes and endophytes than the other identified hosts; *Corallina mediterranea*, *C. officinalis*, *Gelidium crinale*, *Codium* sp., *Jania rubens*, *Pterocladiella capillacea*, *Cladophora pellucida*, *Ulva intestinalis*, and *Petalonia fascia*. This may be due to the fact that there is a difference on the species level till the varaity degree from the point of view of the type and quantity of the secreted metabolites [47]. For instance, *Ulva* is a good source of vitamin B, proteins, minerals (calcium, potassium, magnesium, sodium, copper, iron, and iodine), and dietary fibers [48].

Seaweeds have a rich diversity of associated microorganisms compared with other multicellular organisms. Many studies investigated different algae from different locations all over the world and at varying depths; some of them investigated more than one type of algae, such as green, red, and brown algae [49], and others investigated only one type [50]. Furthermore, they also recorded the phenotypic characterization and physiological properties of associated bacteria with these seaweeds, and they differentiated between them according to these properties. They also categorized them as epiphytes and endophytes [51].

In accordance with our data, Ibrahim et al. [4] studied the algal-associated bacteria and reported similar results; they determined thirteen algal samples collected during spring (April 2012) at six sites: Abu-Qir Bay, El Silsila, Eastern Harbor, El Anfoushy, Western Harbor, and El Dekheila Harbor along the Alexandria coast with depths of 0.5 to 1 m. The thirteen algal samples come from eight different species, grouped into three groups: green, red, and brown. They are *Ulva compressa*, *Ulva fasciata*, *Ulva lactuca*, *Colpomenia sinuosa*, *Corallina officinalis*, *Gelidium crinale*, *Pterocladiella capillacea*, and *Grateloupia doryphora*. The last one was recently introduced to the Egyptian Mediterranean Sea and re-examined by Rodriguez-Prieto et al. [52] and named *Grateloupia gibesii*. It is now thought to be *Phyllymenia gibbesii* (Harvey) [53]. Nine isolates were found in algal samples, each with unique characteristics. The first isolate, A1, was a rod-shaped Gram-ve with motile cells capable of growing in aerobic conditions with temperatures ranging from 20 to 40 ℃. Its growth was reported at different pHs ranging from 5 to 10 and NaCl 1–10%. It was positive for citrate utilization. The second isolate, A2, was the same as A1, except for being non-motile, a restricted aerobe with growth temperatures ranging from 30–37 ℃, a pH ranging from 6 to 9, and NaCl ranging from 1 to 7% and not utilizing citrate. The isolate A3 was the same as A2, except for the growth temperature, which ranged from 20 to 37 ℃ at pH 6–9 and NaCl 0–3%. The isolate A4 was the same as A3, with a growth temperature range of 20–40 ℃ at pH 6–8 and NaCl 0–4%. The isolate A5 was the same as A4, with a growth pH of 5–9 and NaCl of 0–6%. The cells of isolate A6 were characterized as A5 with growth at pH 5–9 and NaCl 1–10%. It was positive for tween80 hydrolysis. Isolate A7 appeared as A6 with growth at pH 4–8 and NaCl 0–6%. The isolate A8 was the same as A7, with growth at pH 5–10 and NaCl 4–10%. It was positive for the Voges-Proskauer test. It produced acids from glucose, maltose, mannitol, sucrose, and lactose. The isolate A9 was characterized as A8 with growth at pH 4–10 and NaCl 1–6%.

In the current study, it was detected that a higher number of epiphytic and endophytic bacteria were isolated from green and red algae; definitely, the percentage of higher isolates was from green rather than red and brown algae. On surface colonization, non-pigmented bacterial isolates were found dominant in most of the seaweeds utilized in this investigation. Epiphytic and endophytic bacteria from marine macroalgae have been well studied in reference to their ecological importance with host organisms with a dominance of Gram-negative bacteria [21]. On the contrary, in the present study, 49 Gram-positive bacterial isolates were isolated in comparison to 16 Gram-negative. Bacteria associated with the species *Ulva lactuca* were dominant, with 17 isolates, followed by other species such as *Corallina mediterranea* (9 isolates) and *Corallina officinalis* (7 isolates). Ravisankar et al. [54] reported that the surface of the brown algae *Hypnea valentiae* and *Padina tetrastromatica* contained a greater number of non-pigmented bacterial colonies, which is different from our study, wherein 17 non-pigmented isolates were obtained from *Ulva lactuca*. Similar observations to our results were detected in Tunisian waters, where 17 isolates were obtained from the thallus of *Ulva intestinalis* [55] and 10 isolates were reported from *Ulva lactuca* in Fiji waters [56]. In our study, forty isolates (62%) of the total 65 isolates showed a broad spectrum of enzymatic activity, with seventeen isolates (26%) having amylolytic activity, fourteen isolates (21%) producing caseinase enzyme, five isolates secreting alginase enzyme (8%), and twenty-six isolates (40%) having cellulolytic activity. In contrast to our results, Ibrahim et al. [4] isolated 100% of the associated bacteria from the algae with catalase activity and 77% with oxidase activity. On the other hand, 22% of the isolated bacteria associated with seaweeds were reported to have starch and gelatin hydrolysis.

## Conclusion

In this study, we shed light on the variety of algal-associated bacteria in four marine sites, even if the algae belong to the green, red, or brown group, and we clarify the difference between epiphytic and endophytic bacteria isolated either from the same or different algae. We clarify the phenotypic properties of the isolated algal associated bacteria and its enzymatic activities against some substrates, also our study stand out the optimum environmental conditions such as salinity, pH, temperature that achieve the best growth of the bacterial isolates, also we explain with statistical analysis the effect of supplying the nutrient medium by algal extract on bacterial growth. There is limited research on eukaryotic-bacterial interactions mediated by cross-kingdom signals. For instance, Indole-3-acetic acid (IAA), a plant hormone, mediates plant-bacteria relationships and can affect bacterial physiology. However, little is known about IAA’s functions in aquatic interactions between algae and microbes, as signaling molecules regulate algal symbioses. Therefore, it is a recommendation for future work.

## Data Availability

No datasets were generated or analysed during the current study.
